# Left atrial strain in neonates with congenital diaphragmatic hernia and length of stay in pediatric intensive care unit

**DOI:** 10.3389/fped.2024.1404350

**Published:** 2024-06-04

**Authors:** Katarina Övermo Tydén, Carmen Mesas Burgos, Baldvin Jonsson, Felicia Nordenstam

**Affiliations:** ^1^ Department of Women’s and Children’s Health, Karolinska Institutet, Stockholm, Sweden; ^2^ Pediatric Cardiology Unit, Karolinska University Hospital, Stockholm, Sweden; ^3^ Department of Pediatric Surgery, Karolinska University Hospital, Stockholm, Sweden; ^4^ ECMO Centre, Karolinska University Hospital, Stockholm, Sweden

**Keywords:** CDH, cardiac function, diastolic function, neonatal, left atrial strain, congenital diaphragmatic hernia, systolic function, left ventricle strain

## Abstract

**Introduction:**

The role of cardiac left ventricle (LV) dysfunction in children with congenital diaphragmatic hernia (CDH) has gained increasing attention. The hernia allows abdominal mass to enter thorax and subsequently both dislocating and compressing the heart. The pressure on vessels and myocardium alters blood flow and may interfere with normal development of the LV. A dysfunctional LV is concerning and impacts the complex pathophysiology of CDH. Hence, assessing both the systolic and diastolic LV function in the newborn with CDH is important, and it may add value for medical treatment and prognostic factors as length of stay (LOS) in pediatric intensive care unit (PICU). LV strain is considered an early marker of systolic dysfunction used in the pediatric population. Left atrial (LA) strain is an echocardiographic marker of LV diastolic dysfunction used in the adult population. When filling pressure of the LV increases, the strain of the atrial wall is decreased. We hypothesized that reduced LA strain and LV strain are correlated with the LOS in the PICU of newborns with CDH.

**Methods:**

This retrospective observational cohort study included data of 55 children born with CDH between 2018 and 2020 and treated at Karolinska University Hospital, Sweden. Overall, 46 parents provided consent. Echocardiograms were performed in 35 children <72 h after birth. The LA reservoir strain (LASr), LV global longitudinal strain, LV dimensions, and direction of blood flow through the patent foramen ovale (PFO) were retrospectively assessed using the echocardiograms.

**Results:**

Children with LASr <33% (*n* = 27) had longer stays in the PICU than children with LA strain ≥33% (*n* = 8) (mean: 20.8 vs. 8.6 days; *p* < 0.002). The LASr was correlated with the LOS in the PICU (correlation coefficient: −0.378; *p* = 0.025). The LV dimension was correlated with the LOS (correlation coefficient: −0.546; *p* = 0.01). However, LV strain was not correlated to LOS.

**Conclusion:**

Newborns with CDH and a lower LASr (<33%) had longer stays in the PICU than children with LASr ≥33%. LASr is a feasible echocardiographic marker of diastolic LV dysfunction in newborns with CDH and may indicate the severity of the condition.

## 1 Introduction

Congenital diaphragmatic hernia (CDH) is an anomaly that affects approximately 2.5/10,000 live births (1). The children most severely affected by CDH have long stays in the pediatric intensive care unit (PICU) and a mortality of approximately 50% (2–4). The length of stay (LOS) in the PICU and the need for mechanical ventilation correlates with the severity of the condition and impacts long-term morbidity (5–8).

Evaluating cardiac dysfunction and pulmonary hypertension are important keys to understand the complex pathophysiology of newborn children with CDH. The abdominal content that has migrated into the thorax cavity via the hernia exerts a pressure on the thoracic organs and negatively impact the development of these organs during fetal life. The lungs will be hypoplastic with underdeveloped and abnormal pulmonary vessels resulting in different degrees of pulmonary hypertension after birth. The heart may also be directly affected, left ventricle (LV) hypoplasia is common. The mechanism behind LV hypoplasia is not clear but a combination of reduced pulmonary blood flow, mechanical compression and altered streaming of blood flow through the foramen ovale and the ductus venosus due to mediastinal shift has been suggested (9). After birth an enlarged and pressure loaded right ventricle (RV) with or without dysfunction may affect the left side of the heart directly through decreased preload but also by ventricular interdependence (10) Even with a normally sized LV dysfunction is common in children with CDH and has received increasing attention (11). Patel et al. (11–13) identified LV dysfunction as a risk factor for children with CDH that was correlated with an increased LOS in the PICU, need for extra corporeal membrane oxygenation (ECMO) support and mortality (14, 15). Understanding the interaction between lung, pulmonary vascular, and cardiac functions is vital for optimizing intensive care. Traditional treatment involving administering pulmonary vasodilating drugs to patients with CDH can deteriorate the cardiovascular condition of patients with the fatal combination of LV dysfunction and increased pulmonary arterial resistance (16, 17). In this “perfect storm”, LV dysfunction aggravates right ventricular (RV) dysfunction and pulmonary hypertension due to hypoxia, acidosis, and ventricular interdependence (10, 18). As a result, the child may experience cardiorespiratory failure, resulting in an increased LOS at the PICU or the need for ECMO support. ECMO can be lifesaving for newborns with CDH (19, 20) but has been associated with high mortality and morbidity (21). Echocardiographic assessment of LV function is often challenging in neonates with CDH. The heart is misplaced in the thoracic cavity by the abdominal contents, and the needed echo windows are difficult to access. Traditionally, in neonates, evaluating systolic LV function is less challenging than evaluating diastolic function (22, 23). Alterations of the direction of blood flow through the patent foramen ovale (PFO) can indicate a reduced LV diastolic function, as seen in neonates with pulmonary hypertension (24).

The echocardiographic method strain, cardiac deformation imaging, has become a promising method to evaluate cardiac function, both systolic and diastolic. Left atrial (LA) strain is used to evaluate LV diastolic function in adults (25–27). LA strain is more user-friendly and less angle- and volume-dependent than other LV diastolic echo measurements (26, 27). Studies of the pediatric population have shown that LA strain is a promising marker of LV diastolic function (28, 29). LV strain is an established method to evaluate systolic function in neonates (22, 30).

The aim of this study was to evaluate LV function with echocardiographic measurements of LV and LA strain and LV dimensions in newborn children with CDH and correlation to LOS in the PICU.

## 2 Materials and methods

### 2.1 Study design

This was a retrospective observational cohort study. This retrospective study was part of the larger, on-going CODE-HEART (Congenital Diaphragmatic Hernia Evaluation of the Heart) study, which includes a prospective study of cardiac function in newborns with CDH compared with that of a control group. In this study the retrospective examination was performed on prior echocardiograms that had been saved digitally for clinical purposes. Medical data were retrieved from medical records at Karolinska University Hospital. The systolic and diastolic function were assessed with LV strain (LVS) and Left atrial reservoir strain (LASr) and were tested for correlation with LOS in the PICU. The outcome LOS was divided into two groups, more (>) or less (≤) than 8 days in the PICU, according to Larsen et al. (31).

### 2.2 Study population

Since 2018, the care of children born with CDH in Sweden has been centralized, and Karolinska University Hospital is the larger of two pediatric surgical centers in Sweden offering this specialized care. This study focused on children born with CDH between 2018 and 2020 who were treated at Karolinska University Hospital, Stockholm, Sweden. The families were asked to participate via telephone, email, or postal mail, and participants were included after oral and written consent. Exclusion criteria were not being born in Sweden, no available presurgical echocardiogram, poor-quality echocardiograms, echocardiograms performed after 72 h of age, and the presence of complex congenital heart disease or complex genetic or chromosomal anomalies. The hernia severity was assessed according to prenatal measurements of the lung size, observed-to-expected lung-to-head ratio (O/E LHR), and postnatal defect size, measured as the need for a patch (32).

### 2.3 Echocardiography

All echocardiographic exams were reviewed retrospectively. The exams were performed between 2018 and 2020 by different pediatric cardiologists and stored digitally in SyngoDynamics version VA40D (Siemens Healthineers, Erlangen, Germany). Echocardiographic exams performed before the surgical intervention and before 72 h of age were reviewed by a senior consultant pediatric cardiologist (KÖT) who also performed all measurements and calculations and was blinded to the severity and LOS. Echocardiograms with poor quality and that were inadequate for strain measurements were excluded, according to the guidelines of the EACVI/ASE/Industry Task Force to standardize deformation imaging (33). Intraobserver variability tests were performed to reduce subjective data, ensure reproducibility, and validate the observations. Strain analyses were performed using the TomTec version TTA2 (TOMTEC Imaging Systems GmbH, Unterschleissheim, Germany). The echocardiographic parameters assessed were the left ventricular end-diastolic dimension (LVED) measured in the apical four-chamber view, *z*-score according to the Detroit data, LVS, LASr, direction of blood flow through the PFO, and direction of blood flow through the arterial duct (DA). For all echocardiographic parameters the mean of three measurements was used for analysis.

**Figure 1:**
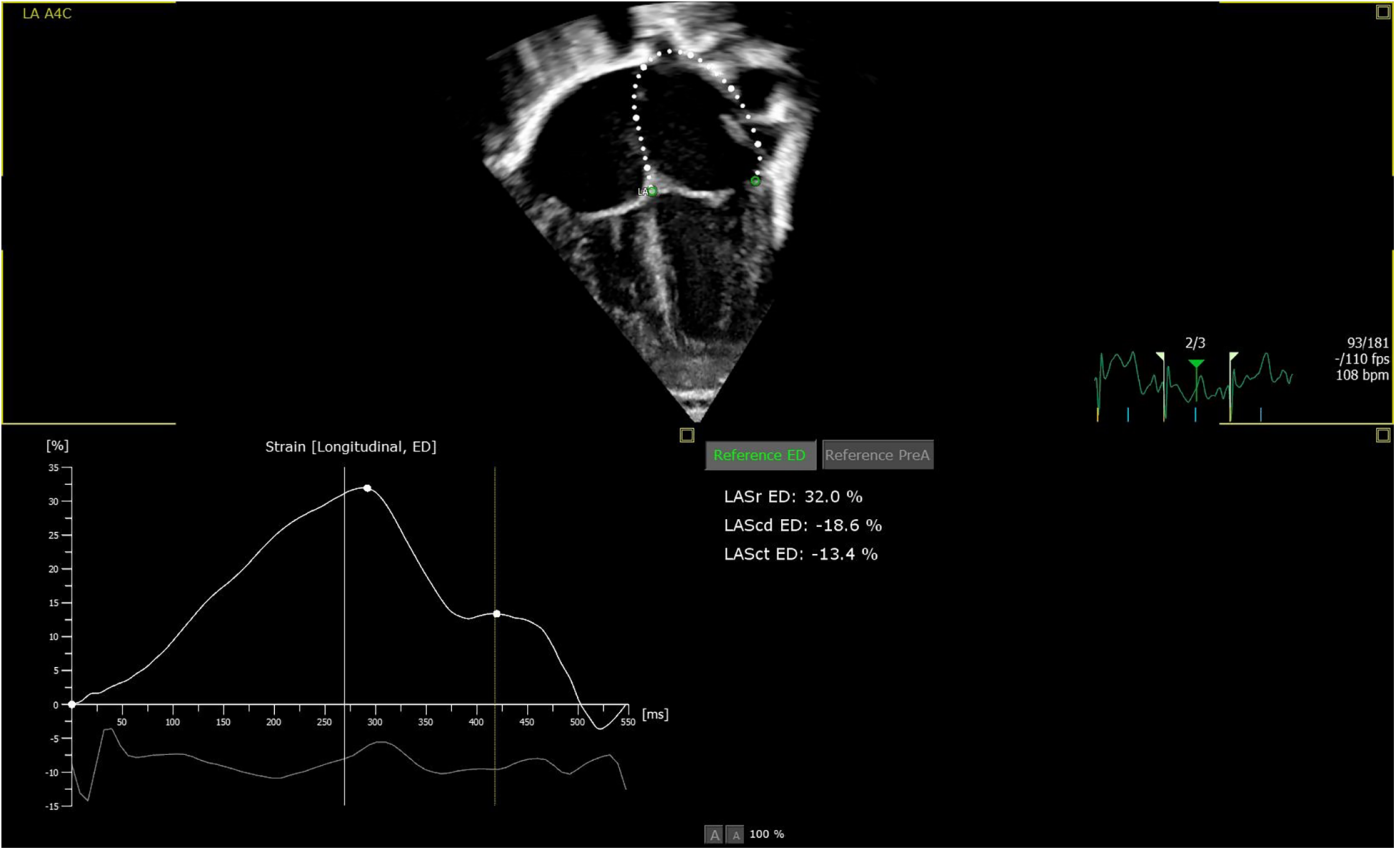
Picture of apical four chamber view: software automatically measuring LASr, defining the endocardial border as region of interest, excluding the pulmonary veins and/or LA appendage orifice.

For strain analyses, 2D speckle tracking strain analyses were used. The endocardial contour was initially tracked automatically by the software defining a region of interest and adjusted manually, as needed. Both LA and LV strain were measured in one plane from 2D apical four-chamber view (34, 35). LVS was measured as peak left ventricular global longitudinal strain and presented in negative percentage the more negative values indicate better LV function. Left atrial reservoir strain (LASr) is a marker of LV filling and LV diastolic function. Other LA strain measurements as conductive and contractile strain have not shown as strong correlations to LV dysfunction as LASr and were accordingly not analyzed in this study (27, 36). When measuring LASr, the pulmonary veins and/or LA appendage orifice were excluded according to the guidelines of the EACVI/ASE/Industry Task force to standardize deformation imaging (33) (Figure 1). To our knowledge, no normal values for the LASr in the neonatal population have been established. Therefore, the chosen cutoff value for a normal LASr was set to >33% in our study, as suggested by Ficial et al. (34). The cutoff value for LVS was −16%, where more negative values than −16% were considered normal according to Patel et al. (11, 22).

### 2.4 Statistical analysis

Categorical data are expressed as frequencies or proportions. Continuous data are expressed as mean ± standard deviation (SD) or median (interquartile range). Non-normally distributed data were analyzed with the Mann-Whitney *U*-test to investigate differences between groups. To compare more than two groups, the Kruskal Wallis test was used. For nominal categorical data, the Chi-square or Fisher's exact test was used. Spearman's non-parametric correlation coefficient was performed to assess linear correlations between variables. A logistic regression model was used to calculate the odds ratio for sex, birth weight, LVED, patch/no patch, direction of blood flow through the DA, and LASr <33%. Uni- and multivariate models were used and compared using forward selection. Intraclass correlation coefficients were used for reliability testing using the two-way mixed effect model. A *p*-value <0.05 was considered statistically significant, and 95% confidence intervals were used. Statistical analyses were performed using SPSS software (version 27; IBM).

### 2.5 Intraobserver variability test

Ten randomly selected echocardiograms, blinded to the observer, were measured twice by the same observer. The interval between measurements was >8 weeks. An intraobserver variability test was then performed using SPSS.

### 2.6 Ethics

This study was approved by the Swedish Ethical Board (Dnr 2020-03543), according to Swedish ethical regulations.

## 3 Results

### 3.1 Study cohort

**Figure 2:**
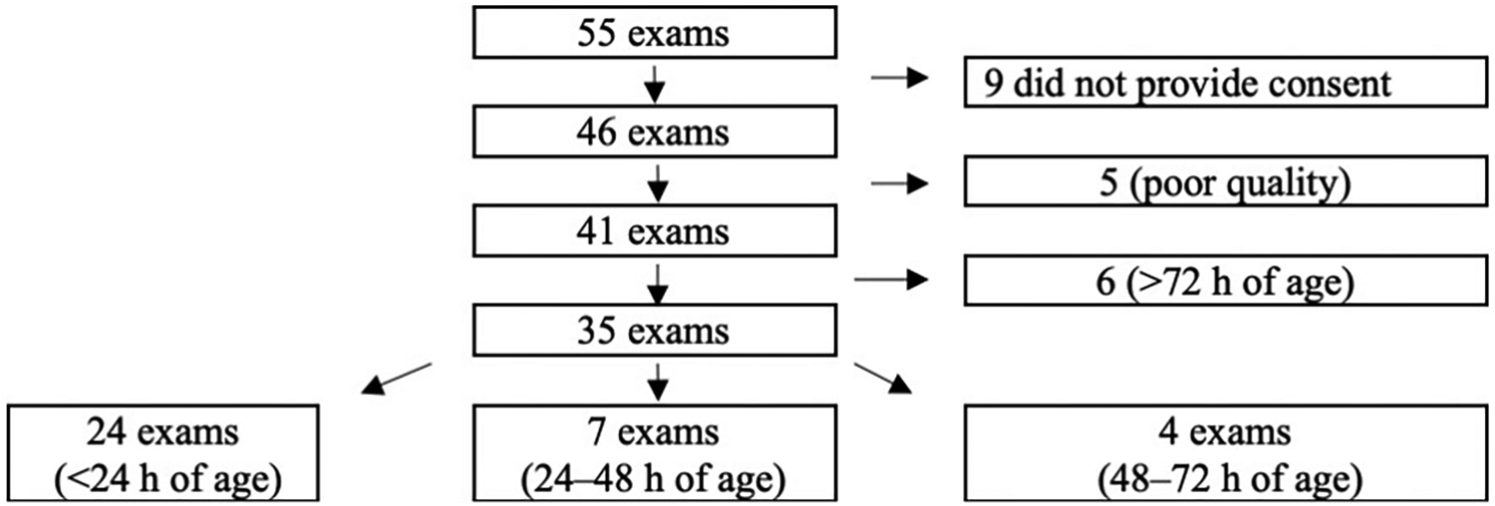
Flow diagram of inclusion and exclusion of the study cohort of children born with congenital diaphragmatic hernia (CDH) and treated at karolinska university hospital from 2018 to 2020.

From 2018 to 2020, 55 Swedish newborns with CDH were treated at Karolinska University Hospital. Overall, 46 caregivers provided consent to participate, and 35 cases were included in the study. Reasons for exclusion are displayed in Figure 2.

Demographic characteristics of participants are shown in Table 1. Among the included cases, no children had complex congenital heart disease (CHD); six children were diagnosed with minor CHD, including atrial septal defects (ASD), ventricular septal defects (VSD), and left superior vena cava (SVC); and one child was born with omphalocele. No children were diagnosed with associated genetic disorders or chromosomal anomalies. Five children received VA (venoarterial) ECMO treatment, 3 echocardiograms were performed on ECMO. Four of the included children required dialysis during their PICU stay or ECMO treatment, all echocardiograms included were performed before dialysis was initiated the dialysis was a part of the intensive care treatment. None of the cases had a primary nephrological diagnosis. At the time of the echocardiograms during the first 72 h, five neonates had on-going Milrinone infusion and 16 had on- going support with inotropes. The inotropes used were noradrenalin (*n* = 10), adrenaline (*n* = 1, vasopressin (*n* = 3), dopamine (*n* = 3), and dobutamine (*n* = 1). A combination of inotrope drugs was used in three cases. In one case it was not possible to find out which inotrope drug that was used. A total of 34 neonates were intubated and on mechanical ventilation when the echocardiograms were performed. Ten neonates had on-going iNO treatment while the echocardiograms were performed.

**Table 1:** Demographic data and CDH characteristics of all cases and the LASr <33% and LASr ≥33% groups.

Subgroup, *n*	All cases	LASr <33%	LASr ≥33%	*p*-value
Number of cases, *n*	35	27	8	
Gestational age, median, range (weeks)	(31 + 0–41 + 0)	31 + 0–41 + 0	36 + 0–41 + 0	0.396
Sex, male, *n* (%)	23 (64)	16 (59)	7 (87.5)	0.216
Birth weight, (kg) mean (range)	2.86 (1.5–3.7)	2.8 (1.5–3.7)	3.0 (2.3–3.65)	0.363
Prenatal diagnosis, *n* (%)	20 (57)	17 (63)	3 (37)	0.246
O/E LHR, *n* (range %)	18 (20–90)	15 (20–90)	3 (50–65)	0.250
Left sided, *n* (%)	26 (74)	21 (77.8)	5 (62.5)	0.396
CHD complex, *n* (%)	0	0		
CHD minor, *n* (%)	5 (14)	4 (14.8)	1 (12.5)	1.0
Genetic anomaly, *n* (%)	0	0	0	
CDH study group stage, *n* (%)				0.223
A	7 (20)	4 (14.8)	3 (37.5)	
B	19 (54)	14 (51.9)	5 (62.5)	
C	5 (14)	5 (18.5)	0	
D	4 (11)	4 (14.8)	0	
Patch repair, *n* (%)	17 (48.5)	15 (55.6)	2 (25.0)	0.228
Liver in chest, *n* (%)	12 (34.3)	9 (33.3)	3 (37.5)	1.0
Survival (28 days), *n* (%)	33 (94.3)	25 (92.6)	8 (100)	1.0
ECMO, *n* (%)	5 (14)	5 (18.5)	0	0.315

### 3.2 Echocardiography

The mean LASr for the whole group of CDH children was 27.4% (SD 10.5). The mean LVS for the whole group of CDH children was −16.9% (SD 4.2). The LASr was correlated with the LOS in the PICU (correlation coefficient: −0.378; *p* = 0.025) but the LVS and LOS were not correlated (correlation coefficient: 0.115; *p* = 0.510). When echocardiograms performed on ECMO support were excluded (*n* = 3) from the analysis, the correlation remained significant (correlation coefficient: −0.428; *p* = 0.015).

**Figure 3:**
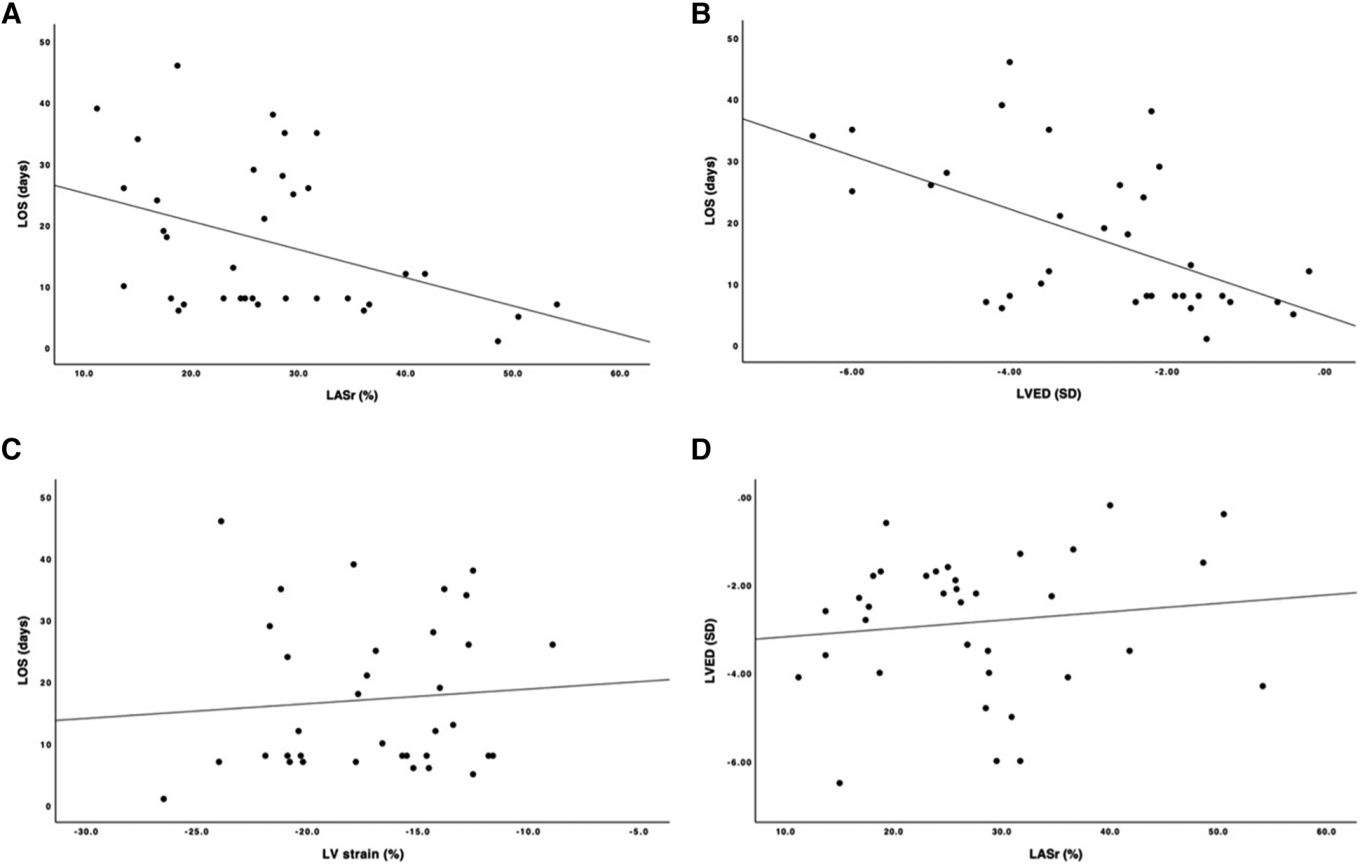
Scatter plot with correlation line. (**A**) y-axis length of stay, LOS (days); x-axis, left ventricular end- diastolic dimension, LVED (SD). (**B**) y-axis, LOS (days); x-axis, left atrial reservoir strain, LASr (%). (**C**) y-axis, LOS (days); x-axis, left ventricular strain, LV strain (%). (**D**) y-axis, LVED (SD); x-axis, LASr (%).

The LVED was correlated with the LOS (correlation coefficient: −0.546; *p* = 0.01), but the LASr and LVED were not correlated (correlation coefficient: 0.109; *p* = 0.532) (Figure 3).

**Figure 4:**
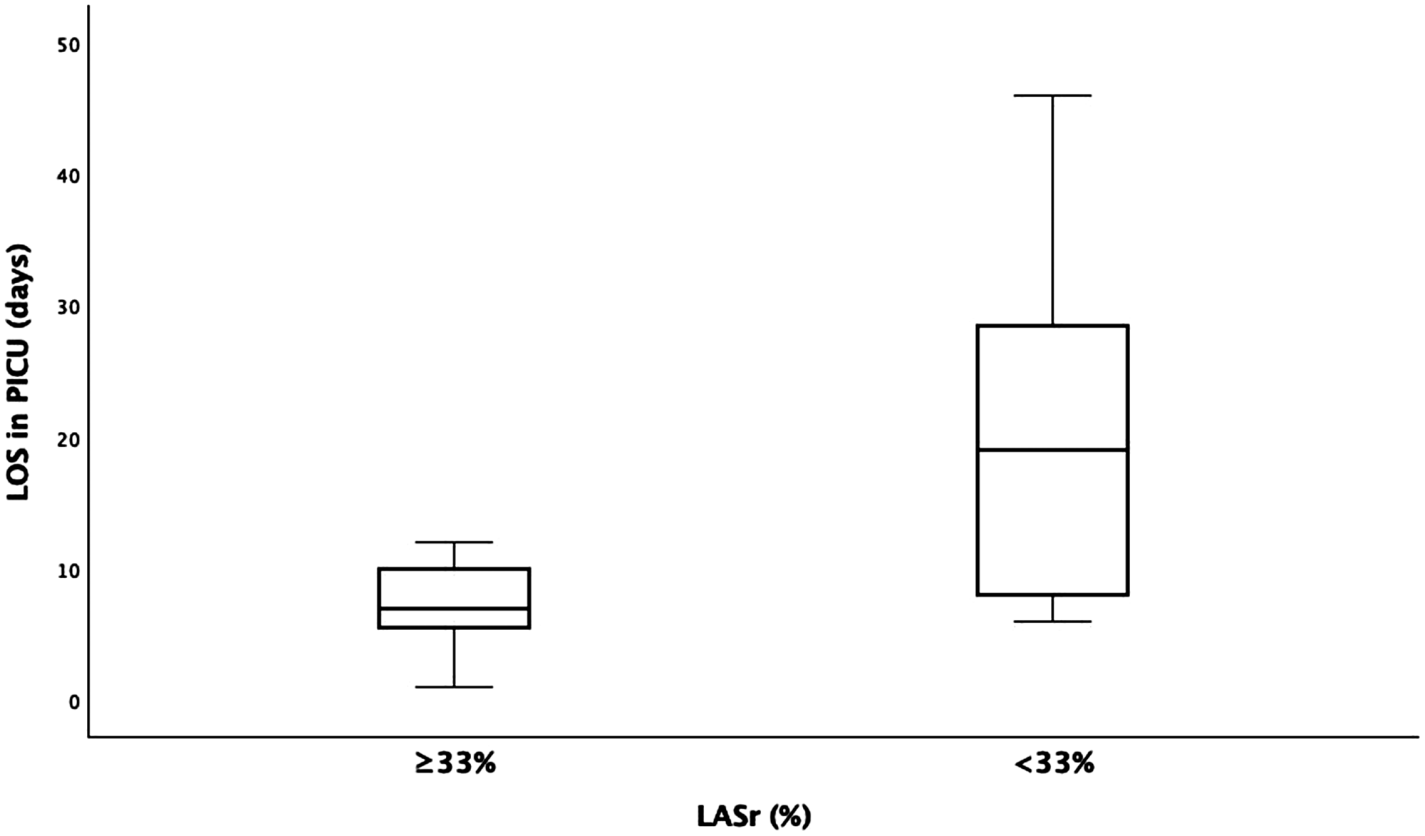
Left atrial reservoir strain, LASr (<33% and >33% groups) compared to the length of stay (LOS) in the pediatric intensive care unit (PICU). y-axis, days; x-axis, LASr strain.

The LASr measurements were divided into two groups: ≥33% (*n* = 8) or <33% (*n* = 27). In our study, measurements >33% were considered normal. These two groups were compared regarding the LOS in the PICU. The group with LASr <33% had a significantly longer PICU stay than the group with LASr ≥33% (20.8 vs. 8.6 days; *p* < 0.002) (Figure 4).

**Figure 5:**
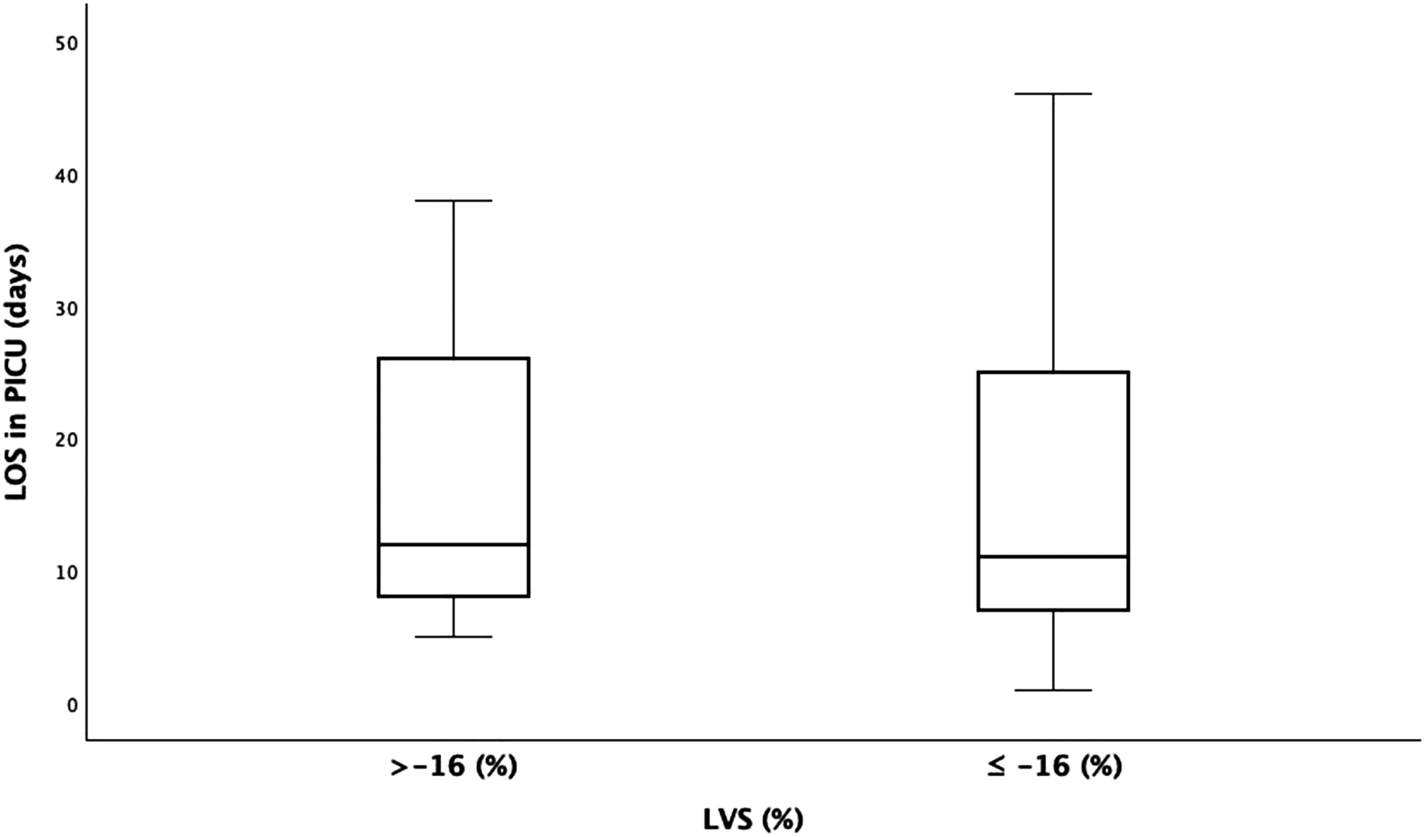
Left ventricle (LV) strain (<−16% and >−16% groups) compared to the length of stay (LOS) in the pediatric intensive care unit (PICU). y-axis, days; x-axis, LV strain.

The LVS measurements were divided into two groups, > or ≤−16%, based on the cutoff value suggested by Patel et al. (11) The LOS in the PICU were not significantly different between the two groups (18.1 vs. 17.92 days; *p* = 0.961) (Figure 5).

Signs of pulmonary hypertension were assessed based on the direction of the blood flow through the DA. Of the cases, 29 (82%) had an open DA, 8 (28%) had a strict right-to-left shunt, and 21 (72%) had a bidirectional shunt. The groups showed no differences regarding the LOS (11.7 vs. 16.2 days; *p* = 0.200).

The use of inhaled nitric oxide (iNO) treatment (*n* = 16) was correlated with increased LOS in the PICU (11.0 vs. 26.4 days; *p* < 0.001).

Of the cases, 33 (88.5%) had PFO, 2 (5.7%) had a right-to-left shunt, 25 (71.4%) had a left-to-right shunt, and 4 (11.4%) had a bidirectional shunt. The groups had no significant differences when compared to the LOS in the PICU (*p* = 0.587) or LASr (*p* = 0.838).

Logistic regression analysis was used to analyze the association between the LOS in the PICU and LASr, after adjusting for LVED, sex, birth weight, and patch/no patch.

After adjusting for possible confounders, the association between the LASr and LOS in PICU remained. [OR = 0.9 (0.817–0.995); *p* = 0.039].

### 3.3 Intraobserver test

The intraobserver variability test showed an intraclass correlation coefficient for the LASr of 0.94, which was considered excellent. For the LVS, the correlation coefficient was 0.73, which was considered acceptable.

## 4 Discussion

This study demonstrated a correlation between a reduced LA strain, assessed as LASr and a longer stay in the PICU in newborn children with CDH. In addition, we observed a correlation between a smaller LV dimension (LVED) and a longer stay in the PICU. However, the LV dimensions and LASr were not correlated. After adjusting for confounders, including LV dimensions, the association between the LASr and LOS remained. Smaller LV dimensions have been identified as contributing factors to the severity of CDH in newborns by Chandrasekharan et al. and Patel et al. (3, 11). However, Cruz-Lemini et al. could not verify these finding (37).

Our study suggests that LA strain and LV dimensions are risk factors of severity in children born with CDH. In adults, measuring the LA strain is a recognized method to evaluate LV diastolic dysfunction (27, 38). However, in neonatal care, few studies have reported on the use of LA strain as a diastolic function parameter, and no established normal data is available. Based on our findings, we suggest that reduced LASr is a useful marker for diastolic dysfunction in neonates. Earlier studies have shown that LASr is a more sensitive and accurate parameter to diagnose diastolic dysfunction compared to other LA strain measurements as conduit and contractile LA strain (26, 27, 36). In this study, only LASr was measured as a surrogate marker for LV diastolic dysfunction.

The LVS was not correlated with the LOS in the PICU. However, the LVS was reduced among newborns with CDH, when compared to normal values (39). LVS is suggested as a marker for systolic function in neonates (40). The decreased LVS in neonates with CDH in this study align with the findings of systolic dysfunction in neonates with CDH described in earlier studies (11, 41).

One factor that may affect both the LOS in the PICU and LASr strain is the degree of pulmonary hypertension. Our echocardiograms were all performed early in life (<72 h of age) and may be influenced by a delayed transition of the neonatal circulation. The assessment of pulmonary hypertension includes several echocardiographic parameters and must be linked together with the assessment of LV function and clinical status (12). The direction of blood flow through the arterial duct may indicate presence of pulmonary hypertension. We found no correlation between the LOS or LASr and the direction of flow in the arterial duct in our study population; however, pulmonary hypertension needs to be assessed more thoroughly with different echocardiographic measurement methods before this finding has bearing. Further, in this study, the use of iNO treatment was correlated with the LOS in the PICU. Since iNo is used to treat pulmonary hypertension, this might imply that pulmonary hypertension influences LOS. However, another possible explanation is that severely ill children with CDH receive iNO regardless of LV dysfunction, and the increased preload to the dysfunctional LV may aggravate the situation, leading to longer stays in the PICU. Thus, if the diastolic dysfunction is unknown, administering pulmonary vasodilating drugs can cause children with CDH to experience cardiorespiratory failure (17). Hence, we suggest that future studies assess the LASr and LVS before considering iNO treatment.

No correlations were observed between either the LOS in the PICU or LASr and the direction of blood flow through the PFO. Similarly, earlier studies have shown that the direction of blood flow through the PFO is not correlated with cardiac function (11, 14). Nevertheless, assessing the PFO is still important, and the gradient over the PFO may be more important than the direction of the flow when assessing diastolic dysfunction in neonates with CDH. Unfortunately, as our study was a retrospective, measuring the gradient over the PFO was not possible.

### 4.1 Limitations and strengths

This study has several limitations, the study was retrospective; thus, the echocardiographic exams were not performed according to a pre-defined research protocol, and not all required echocardiographic assessments were eligible. The assessment of pulmonary hypertension was limited by the retrospective analysis, with few cases displaying the typical signs of pulmonary hypertension as tricuspid regurgitation, flattening of the septum or right to left shunt in arterial duct. In addition, the exams were performed by different pediatric cardiologists. The frame rate was not considered, which might have affected the strain measurements. The age of the children when the echocardiograms were performed also differed. Several exams were excluded, due to poor quality and being performed after 72 h of age, resulting in a small sample size, which may have impacted the results. Another limiting factor was the lack of a control group. Furthermore, the absence of normal values for LA strain in the neonatal period was a limitation. Comorbidities could also affect the LOS in the PICU. Minor congenital heart disease is unlikely to affect the LOS in the PICU and should therefore not have influenced the results. One child was diagnosed with a minor omphalocele, and we cannot rule out the possibility that the omphalocele impacted the LOS (42). In Sweden, the termination of pregnancy (TOP) rate for prenatal CDH is approximately 30% (2), which may have resulted in a selection bias for less severe CDH cases among the newborns with CDH.

This study also had strengths. The same experienced cardiologist reviewed all echocardiograms and performed all measurements in a standardized setting. The experienced cardiologist reviewing the examinations had no personal involvement in the initial care of the included study participants and was blinded to the outcome during analysis. The echocardiograms were stored on a server that was separate from the medical records, and echocardiograms and medical journals were reviewed separately. The intraobserver variability was excellent in the LASr correlation test and acceptable in the LVS correlation test. LASr is a user-friendly echocardiographic measurement that can be used in clinical practice, even when conditions to perform echocardiography are not optimal. Furthermore, the software used was not vendor dependent (TomTec), and all echocardiographic exams were performed by trained cardiologists in the same pediatric cardiology unit and stored on the same server system. All children were cared for and managed at Karolinska University hospital; therefore, few cases were missing medical data. The standardization of treatment at Karolinska University Hospital of children born with CDH before, during, and after birth enhances the robustness of the study findings.

### 4.2 Conclusion

In our study population, we identified a correlation between a reduced LA strain and a longer stay in the PICU, where the echocardiographic measurement of LASr reflects the diastolic cardiac function of the left ventricle. LASr strain is a feasible echocardiographic parameter and may be used as a marker of severity in children born with CDH.

## Data Availability

The raw data supporting the conclusions of this article will be made available by the authors, without undue reservation.
